# Every second counts: digital and analogue mammography - comparison of reading times at the Queen Elizabeth Breast Screening Unit, Gateshead, UK

**DOI:** 10.1186/bcr2971

**Published:** 2011-11-04

**Authors:** GD Bristow, AJ Potterton, LG Lunt

**Affiliations:** 1 The Queen Elizabeth Hospital Breast Screening Unit, Gateshead, UK

## Introduction

In the NHS Breast Screening Programme (NHSBSP) there is a transition to digital mammography following recommendations made by the Cancer Reform Strategy [1]. A number of US studies have demonstrated that the time taken to interpret digital mammography is longer than that for analogue [2,3]. There are no published data about this from the NHSBSP.

## Methods

Over a 2-month period, 11 readers were timed in their interpretation of batched analogue or digital mammograms. These were either hung on a multiviewer or preloaded onto Sectra PACS. Previous images were not digitised. A total of 396 batches were included in the analysis (unpaired *t *test), 330 digital and 66 analogue.

## Results

It takes more time to report a digital mammogram compared with analogue (40 ± 1 vs. 35 ± 2 seconds, *P *< 0.05). There is no difference in the time taken to report prevalent screens between the analogue and digital groups (34 ± 7 vs. 39 ± 2 seconds). The incident screens were quicker to interpret as analogue.

## Conclusion

Our data support the hypothesis that digital interpretation is slower than analogue (albeit by 5 seconds) but in the absence of needing to compare with previous images there is no difference between the two modalities.
